# Accelerating Fibre Orientation Estimation from Diffusion Weighted Magnetic Resonance Imaging Using GPUs

**DOI:** 10.1371/journal.pone.0061892

**Published:** 2013-04-29

**Authors:** Moisés Hernández, Ginés D. Guerrero, José M. Cecilia, José M. García, Alberto Inuggi, Saad Jbabdi, Timothy E. J. Behrens, Stamatios N. Sotiropoulos

**Affiliations:** 1 Department of Computer Science, University of Murcia, Murcia, Spain; 2 Department of Computer Science, Catholic University of Murcia, Murcia, Spain; 3 Basque Center on Cognition, Brain and Language, San Sebastian, Spain; 4 Centre for Functional MRI of the Brain (FMRIB), University of Oxford, Oxford, United Kingdom; University of Minnesota, United States of America

## Abstract

With the performance of central processing units (CPUs) having effectively reached a limit, parallel processing offers an alternative for applications with high computational demands. Modern graphics processing units (GPUs) are massively parallel processors that can execute simultaneously thousands of light-weight processes. In this study, we propose and implement a parallel GPU-based design of a popular method that is used for the analysis of brain magnetic resonance imaging (MRI). More specifically, we are concerned with a model-based approach for extracting tissue structural information from diffusion-weighted (DW) MRI data. DW-MRI offers, through tractography approaches, the only way to study brain structural connectivity, non-invasively and in-vivo. We parallelise the Bayesian inference framework for the ball & stick model, as it is implemented in the tractography toolbox of the popular FSL software package (University of Oxford). For our implementation, we utilise the Compute Unified Device Architecture (CUDA) programming model. We show that the parameter estimation, performed through Markov Chain Monte Carlo (MCMC), is accelerated by at least two orders of magnitude, when comparing a single GPU with the respective sequential single-core CPU version. We also illustrate similar speed-up factors (up to 120x) when comparing a multi-GPU with a multi-CPU implementation.

## Introduction

Having effectively reached a limit in the improvement of the single-core frequency of central processing units (CPUs), parallel computing has become the method of choice for applications with high computational demands. Even if parallelisability is not always guaranteed, it can be potentially achieved at a high or low level scale for many applications [1]–[3]. For heavily parallelisable tasks, the performance improvement is an increasing function of the number of available computing cores; the more cores are available, the higher the speedups that can be achieved compared to sequential counterpart versions.

Modern Graphics Processing Units (GPUs) are massively parallel processors that contain hundreds of computing cores. Even if the instruction sets of these cores are much simpler than the respective of CPUs [4], GPUs are capable of supporting thousands of threads running in parallel, reaching (at least theoretically) peak performances up to a TeraFLOP (A trillion floating point operations per second). Many general-purpose applications have been successfully ported to these platforms, obtaining considerable accelerations[5]–[7]. Particularly for Magnetic Resonance Imaging (MRI), the potential and increased performance of GPU-based designs has been clearly illustrated for the computationally-demanding task of image reconstruction [8]–[11].

In this paper, we are concerned with the GPU parallelisation of analysis methods applied for studying brain’s structural connectivity through diffusion-weighted magnetic resonance imaging (DW-MRI) [12], [13]. We illustrate how a GPU-based design and implementation can dramatically improve the performance of one of the most popular approaches for processing this type of data. This approach involves Bayesian inference and parameter estimation through Markov Chain Monte Carlo (MCMC) of the ball & stick model [14]–[16], applied repetitively to volume elements (voxels) of three-dimensional images. A sequential implementation of the algorithm is included into Oxford’s software library (FSL) [17], [18] via the *bedpostX* toolbox. Despite its popularity, a drawback of this toolbox is the long computation time, since depending on the parameters of the MRI acquisition protocol, the analysis of a single dataset can easily take more than 24 hours on a single-core CPU.

The bedpostX toolbox belongs to a family of processing methods that provide unique information for studying brain’s structural connectivity. Traditional neuroimaging techniques cannot provide enough information about the anatomical connections between brain regions. However, with the advent of DW-MRI [19] and variants, such as Diffusion Tensor Imaging (DTI) [20], connectivity analysis has become feasible, non-invasively and in-vivo. Algorithms for mapping the brain connections, collectively termed as tractography methods [21], [22] have opened new possibilities for tackling both neuroscience and neuropathology questions.

BedpostX is one of the major components of FSL’s probabilistic tractography [14], [23] toolbox. We introduce here a parallelisation of the toolbox on NVIDIA GPUs by using the CUDA programming model [24]. We identify two main modules in this application that are studied separately. The first provides a starting point for the MCMC algorithm by fitting the model deterministically, minimising the sum of squared residuals. A data parallelism approach is used for this module. The second module performs the MCMC. We propose a design to increase parallelism, but also avoid long-stall warp serialization. Speed-up factors of up to 124x and 135x are achieved for each of the two modules respectively, giving an overall speed-up of up to 112x in a single GPU compared to the sequential single-core CPU version. We also illustrate an overall speed-up of up to 120x for a cluster of GPUs compared to a CPU cluster version.

The impact of these accelerations cannot be underestimated, as they change our perception on what is computationally feasible for brain anatomical studies. Big databases, comprising of massive and high-resolution datasets are soon becoming available (for instance through the Human Connectome Project [25]). Data analysis within reasonable time frames is a major engineering challenge and studies that reduce computation times by orders of magnitude are assisting towards this direction. Furthermore, more accurate but computationally demanding models [26] could be benefitted from similar approaches. Finally, closer to real-time processing could make probabilistic tractography methods more appealing for clinical practice (as in neurosurgical planning [27]).

The rest of the paper is structured as follows. We start by introducing background concepts on DW-MRI, the ball & stick model and GPU programming. Then, we describe the sequential algorithm that is used by the bedpostX application. We present in detail the parallel version implemented in CUDA. The sequential and parallel implementations are compared using a set of tests. Discussion of results and conclusions are presented in the last section. Preliminary results of this work have been presented before in a short conference paper [28].

## Background

### Diffusion-Weighted MRI and the Ball & Stick Model

Diffusion-weighted MRI is sensitive to the diffusion motion of water molecules. Features of diffusion vary throughout the different brain tissues; white matter, which mostly comprises of neuronal axons, grey matter, which contains mainly cell bodies, and cerebrospinal fluid (CSF)-filled regions. Particularly in white matter diffusion is anisotropic; water diffuses preferably along rather than across the axons [29]. In grey matter and CSF regions diffusion is isotropic, i.e. there is no preference for diffusion along any particular orientation. By applying strong magnetic field gradients in several and different directions, it is possible to map these *Preferred Diffusion Orientations* (PDOs) in each image volume element (voxel), where diffusion is anisotropic. Thus, PDOs provide local fibre orientation estimates, i.e. the major axon orientations within an image voxel [30]. Tractography approaches can then utilise PDOs to reconstruct the underlying brain connections, which are mediated by bundles of neuronal axons [21].

To estimate the PDOs a set of diffusion-weighted magnetic resonance images are needed. Each DW image has a contrast that is sensitive to diffusion motions along a specific direction, the direction of an applied diffusion-sensitizing magnetic field gradient [31]. Many DW images are commonly acquired along *K* different directions to effectively sample the signal on a unit sphere domain.

Various model-based and model-free approaches have been suggested to estimate the PDOs through the DW-MRI spherically-sampled signal in each voxel [32]. A popular approach that also takes care of within-voxel fibre crossings, a common problem in tractography, is the ball & stick model [14], [15]. The approach has been implemented in the bedpostX toolbox of the FSL software (developed by the centre for Functional MRI of the Brain, FMRIB, at the University of Oxford). The ball & stick model explains this signal in each voxel of the brain volume, using a multi-compartment decomposition. It assumes a fully isotropic compartment (the ball) and 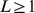 perfectly anisotropic compartments (the sticks). The orientations of these sticks provide the PDOs in a voxel.

Equation 1 shows the signal model when each of the 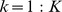 gradient directions is applied:
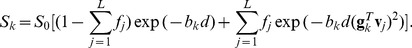
(1)



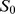
 is a baseline signal without any diffusion weighting; 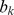 depends on the magnitude and duration of the 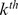 diffusion-sensitizing gradient, 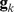 indicates the direction of this gradient, *d* is the diffusivity, and finally 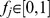 and 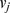 describe the volume fraction and orientation of the 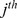 stick (PDO), with:
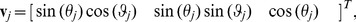
(2)with 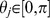 and 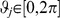. The above model has 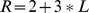 unknown parameters to be estimated.

BedpostX inverts the above model using a Bayesian inference framework. Equation 1 can be used to obtain the likelihood, the conditional distribution of the data measurements given the model parameters. Bayes theorem allows us to calculate the posterior distribution of the parameters given the data 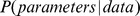. Thus, a distribution is estimated for each of the model parameters 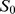, *d*, 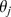, 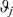 and 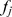, 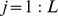. This is performed using a *Markov Chain Monte Carlo (MCMC)* algorithm [33], which is initialized using a *Levenberg-Marquardt* fit of the model to the data [34].

Certain features of the above implementation make it a good candidate for a GPU-based design. These can be summarised into the following: a) Independence between voxels across the three-dimensional brain volume allows voxel-based parallelisation, b) Within each voxel, certain computation steps of data analysis are intrinsically iterative and independent, allowing further parallelisation (for instance, likelihood calculation within the MCMC), c) Relatively simple mathematical operations are needed and these can be handled effectively by the GPU instruction set and d) Memory requirements are moderate during each step of the algorithm.

### The NVIDIA’s GPU Architecture and CUDA Programming Model

**Figure 1:**
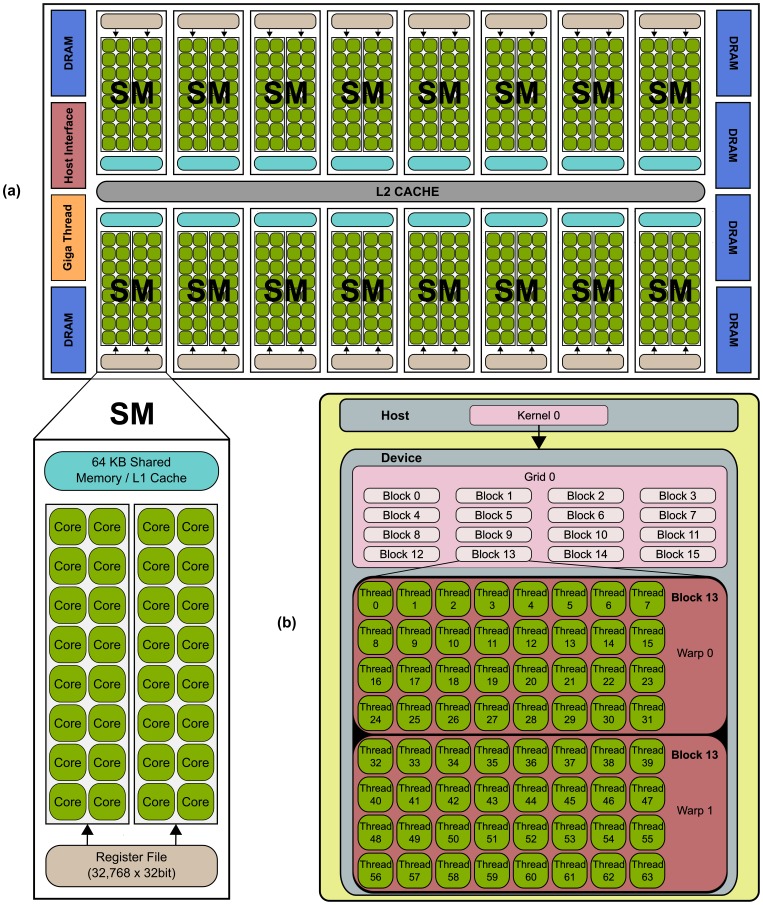
Typical NVIDIA GPU architecture. The GPU is comprised of a set of Streaming MultiProcessors (SM). Each SM is comprised of several Stream Processor (SP) cores, as shown for the NVIDIA’s Fermi architecture (a). The GPU resources are controlled by the programmer through the CUDA programming model, shown in (b).

All NVIDIA GPU platforms from the G80 architecture may be programmed using the CUDA programming model [24], which makes the GPU operate as a highly parallel computing device. Each GPU device is a scalable processor array consisting of a set of SIMT (Single Instruction Multiple Threads) [4], [35] Streaming Multiprocessors (SM), each containing several Stream Processors (SPs) (see Figure 1(a)). The GPU has a global scheduler (Giga Thread) for distributing the work to the SMs and a host interface. Different memory spaces are also available within a GPU, having different latencies, storage capacity and access methods. These memory spaces, ordered from low to high latency are: the register file (32768 32-bit registers per SM in NVIDIA compute capability devices 2.X), the shared memory/L1 cache (64 KB per SM), the L2 cache (768 KB) and the global memory (DRAM, 1 - 6 GB).

The CUDA programming model utilises this architecture and is based on a hierarchy of abstraction layers (see Figure 1(b)). The **thread** is the basic execution unit that is mapped to a single SP.

A **thread-block** or simply block is a batch of threads assigned to the same SM, and therefore share all the resources included in that multiprocessor, such as the register file and shared memory. The threads within a block can communicate through the shared memory. Finally, a **grid** is composed of several blocks which are equally distributed and scheduled across all SMs in a non-deterministic manner.

Threads included within a block are divided into batches of 32 threads called **warps**. The warp is the scheduled unit, so the threads of the same block are executed in a given multiprocessor warp-by-warp in a SIMD (single instruction, multiple data) fashion. The programmer arranges parallelism by declaring the number of blocks and the number of threads per block to use in a specific kernel. To avoid wasting SP resources, the number of threads per block should be a multiple of 32 (i.e. a warp). The maximum number of threads per block since NVIDIA 2.0 compute capability is 1024.

The reader is referred to [35] for a comprehensive overview of the Fermi architecture and to [24] for a comprehensive overview of the GPU programming model.

## Methods

### Description of the Sequential BedpostX Implementation

The input of the bedpostX application is a 4D dataset that represents the DWI brain acquisition of a given subject, with the three dimensions representing location in space (i.e. voxel coordinates) and the fourth corresponding to the *K* diffusion-sensitising gradients applied (i.e. one 3D volume corresponding to each diffusion gradient). The computational demands depend on the size of the dataset, but also on the number *L* of fibre orientations (sticks) to be estimated, as well as on the number *T* of iterations of the MCMC algorithm. The parameter estimation for each voxel location is performed independently and sequentially on a single-core CPU.

**Figure 2:**
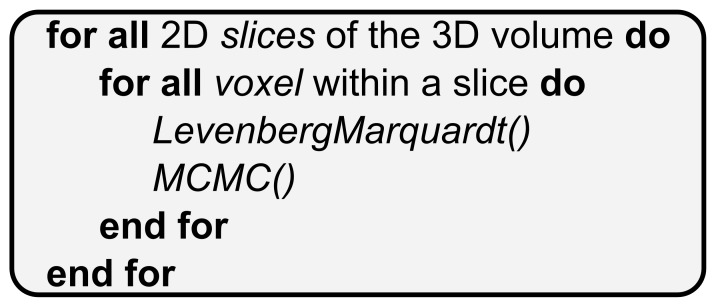
The sequential pseudo-code of *bedpostX.*

Figure 2 summarizes this sequential process, which comprises of two main steps: (1) An initial estimation of the parameters through a Levenberg-Marquardt algorithm, and (2) the estimation of the posterior distribution of the model parameters given the data through the MCMC.

The Levenberg-Marquardt algorithm is based on an iterative numerical optimization procedure that minimizes the sum of squared model residuals [34]. It performs a first, deterministic estimation of the parameters for each voxel and provides a starting point for the MCMC algorithm. The algorithm then proposes in an iterative fashion values for each parameter drawn from Normal proposal distributions (random walk Metropolis).

Whenever a new parameter value is proposed, its posterior probability needs to be evaluated. This involves the calculation of a likelihood term (thus multiple signal calculations using equation 1 are needed) and a prior probability term, that describes our prior belief for the parameters before looking at the data. The proposed parameter values are then accepted or rejected based on a Metropolis acceptance criterion. Therefore, within a MCMC iteration, the step with the highest computational cost is the calculation of the posterior probability value each time a new parameter value is proposed.

### Parallel Design in CUDA

A first straight-forward step towards a parallel design is motivated by the independent nature of the model parameters between voxels. This would entail parallel processing of multiple voxels. In FSL, a similar philosophy is followed [17], [18]. Large groups of voxels (e.g. slices) are fed into different CPU cores and processed independently and sequentially by using a large CPU cluster and the SunGridEngine [36]. This design, however, is based on task parallelism and, thus, it involves heavy tasks assigned to each processor. Task-based parallelism is not theoretically well-suited for GPU programming; within a SM of the GPU, all threads that belong to the same warp will execute the same instruction at a time. Therefore, they cannot execute different tasks, but they can execute the same instruction over different data. A data-based approach can lead to better performance, taking advantage of the thousands of light-weight threads that can run in parallel [37].

Our data-based parallelism approach for this problem is obtained by thinking about how data can be partitioned. We utilise parallelisation in the sub-voxel level. As we have seen, two computational stages run for every voxel (the Levenberg-Marquardt and the MCMC) and these are independent across voxels. These stages are identified as CUDA kernels, which are executed one after the other in a massively parallel way on the GPU.

#### Levenberg-marquardt kernel

**Figure 3:**
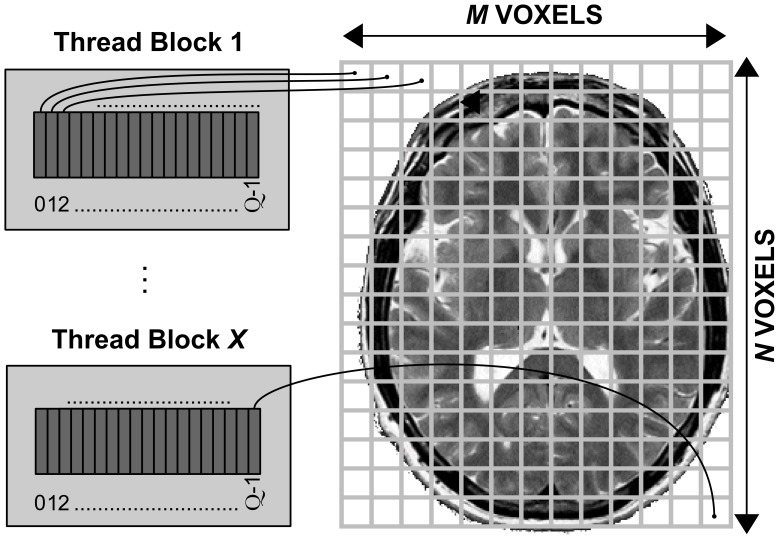
Distribution of resources for the CUDA kernel that performs the Levenberg-Marquardt algorithm. Voxels are assigned to threads of CUDA blocks. Each CUDA block is comprised of 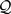 threads and processes 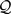 voxels (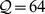 was used in this study).

The first CUDA kernel performs the Levenberg-Marquardt algorithm. This kernel maps each CUDA thread to a voxel (see Figure 3), and it launches as many threads as voxels contained in a particular slice. Because processing of different voxels is totally independent, the threads do not need to synchronize. It is noteworthy that each thread must compute all steps of the Levenberg-Marquardt algorithm using large intermediate structures (the size of the structures depends on the number of parameters to fit and the number of gradient directions *K* of the input dataset). That involves managing many hardware resources and on-chip memories, specifically the registers, which are limited to a maximum number of 64 per thread, at least up to NVIDIA’s Fermi architecture used here. We should point out that for input datasets with a high number *K* of diffusion-sensitising directions, a thread may run out of registers and therefore need to utilise the Global memory. This will inevitably increase latencies each time data have to be read or written. The more recent Kepler architecture [38] supports up to 255 registers per thread and will potentially improve performance.

To achieve a high occupancy of the GPU hardware, while also accounting for the fact that different slices in the brain may have very different number of voxels, we optimised the number of threads per block 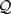 (which as explained before needs to be a multiple of 32 to avoid wasting resources with under-populated warps). The target is to have as many threads as possible per SM (organised in warps) to “hide” latencies that may be induced by Global memory access (while a warp is accessing Global memory, the SM can process another warp). The available number of registers per SM is 32768. If we use the maximum number of registers per thread (minimizing that way the number of Global memory accesses), the maximum number of threads running per SM simultaneously is 512 (32768 registers/64 registers per thread = 512 threads per SM or 16 warps per SM). Choosing how to distribute these threads in blocks of size 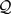 affects performance.

In general, smaller 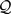 provides greater flexibility to the scheduler to distribute threads better. For instance, let’s imagine the case where 2 SMs are free and there are only 128 voxels (threads) to be processed. If we choose 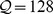 (4 warps) we will use only one of the two SMs (all threads belong to the same block and therefore must be executed by the same SM). But if 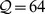 (2 warps) we can use both SMs (2 warps in each SM), therefore achieving greater parallelisation. The minimum number for 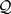 is 32 (to avoid under-populated warps). However, a limitation imposed in NVIDIA 2.X compute capability devices is that any SM can only handle 8 different blocks simultaneously. Therefore, if we set 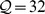 the maximum number of warps simultaneously in a SM are 8 (32 threads * 8 blocks/32 threads of a warp), i.e. half of the maximum warps an SM can handle in our case. Therefore, we set the number of threads to the next minimum number 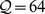 to get the best balance of threads between the different SMs and the best performance for this algorithm in our application.

#### MCMC kernel

Our second CUDA kernel implements the MCMC algorithm. This computation has three main issues: (1) it needs to draw random samples from probability distributions (in this case Normal and Uniform distributions), which implies generation of many random numbers for each voxel, (2) the input of each iteration depends on the output of the previous one, and thus, iterations have to be processed sequentially, and (3) there are dependencies among the different parameters that compose the signal (Equation 1).

**Figure 4:**
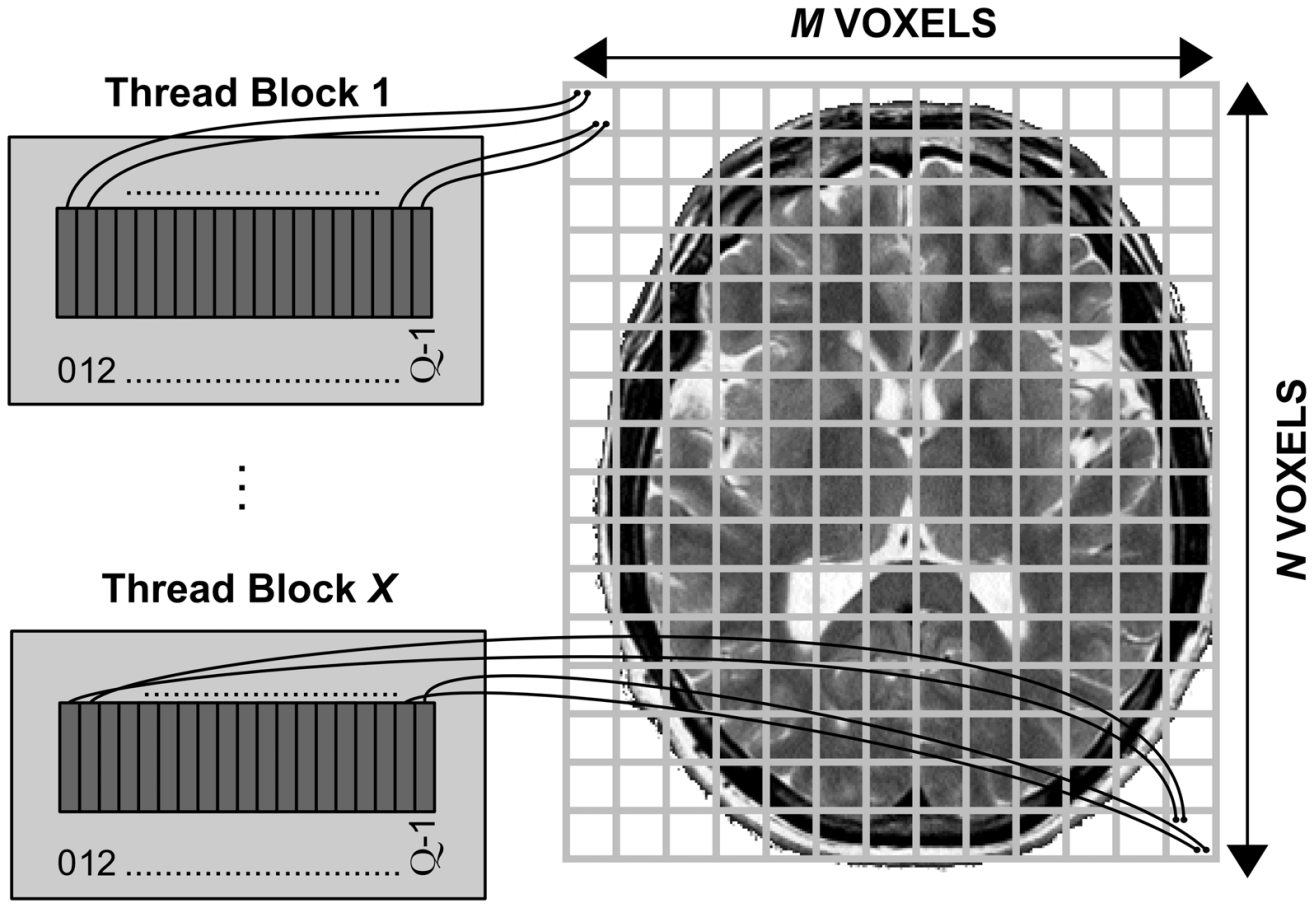
Distribution of resources for the CUDA kernel that performs the MCMC algorithm. Each voxel is assigned to more than one thread within a thread block, so that the likelihood calculation is parallelised. Each CUDA block is comprised of 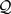 threads and processes only 1 voxel (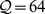 was used in this study).

**Figure 5:**
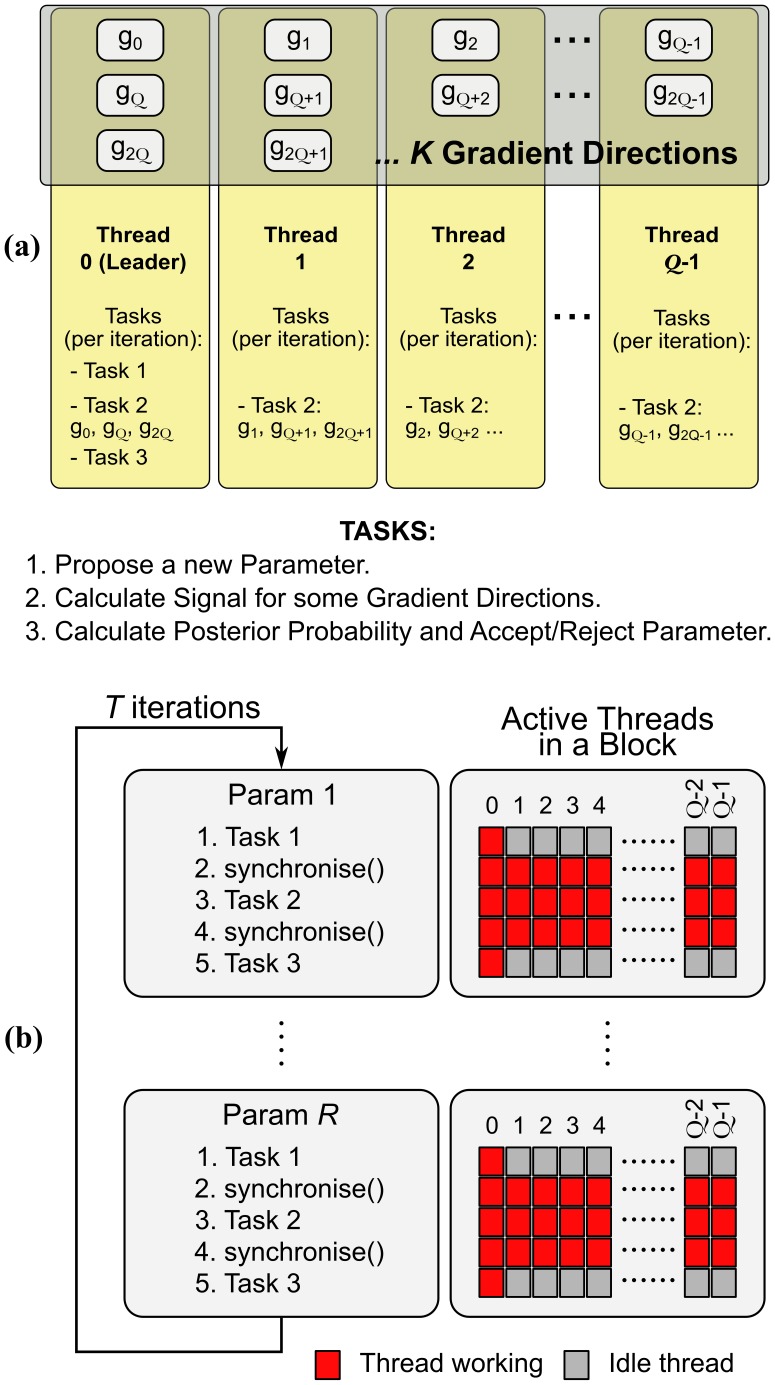
Workflow in the MCMC kernel. (a) Workflow for a single iteration and a single parameter update describing how computation tasks are distributed between the threads of a block (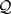) in a case with 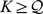 gradient directions. The calculation of the model-predicted signals for the different gradient directions is distributed as evenly as possible between threads within a thread block. The remaining tasks, which are not computationally demanding, are performed by a leader thread, while the rest of threads are waiting. (b) Workflow for a thread block of the MCMC kernel that performs all *T* iterations for all *R* parameters (i.e. for a voxel). Each block has 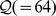 threads. The threads need to be synchronised at certain steps.

The random number generation is computationally expensive. Therefore, we decided to perform the calculation of all random numbers in a separate kernel, before the MCMC kernel execution takes place. Figures 4 and 5 show the underlying design of the MCMC kernel that efficiently addresses (2) and (3) according to the CUDA best practices [39]. Contrary to the Levenberg-Marquardt kernel, each voxel is processed by more than one thread and is assigned to a thread block of size 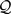. This design allows many, very light-weight threads (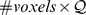 threads) to fully occupy the hardware resources of the GPU.

During a MCMC of *T* iterations (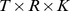) evaluations of Equation (1) and (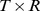) posterior probability evaluations are needed. During each posterior probability evaluation (Figure 5A), the calculations of the model predicted signal for the *K* different diffusion-sensitising gradients are distributed and parallelised between the different threads within the thread block as evenly as possible. When 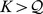, more than one evaluations of Equation (1) are performed by each thread, at a given iteration of the algorithm. As shown in Figure 5A it is possible for some threads to perform one more calculation of Equation (1) than others.

After calculating *K* model-predicted signals, the threads from the same block are synchronised and their results are jointly used through the shared memory of the respective SM, to produce the posterior probability values. In each iteration of the MCMC algorithm, there are some steps (propose a new parameter and calculate the posterior probability to make the decision of whether to accept or reject) undertaken by one thread of the block. While this “leader” thread performs these tasks (Tasks 1 and 3 in Figure 5A), the other threads are waiting. As these tasks are very fast, latencies are minimal. In addition, before each task, all the threads of the block must be synchronised, as there are dependencies across tasks. Figure 5B further illustrates the workflow and pseudo-code with these necessary synchronizations of the MCMC algorithm for a CUDA thread block processed in the GPU.

**Figure 6:**
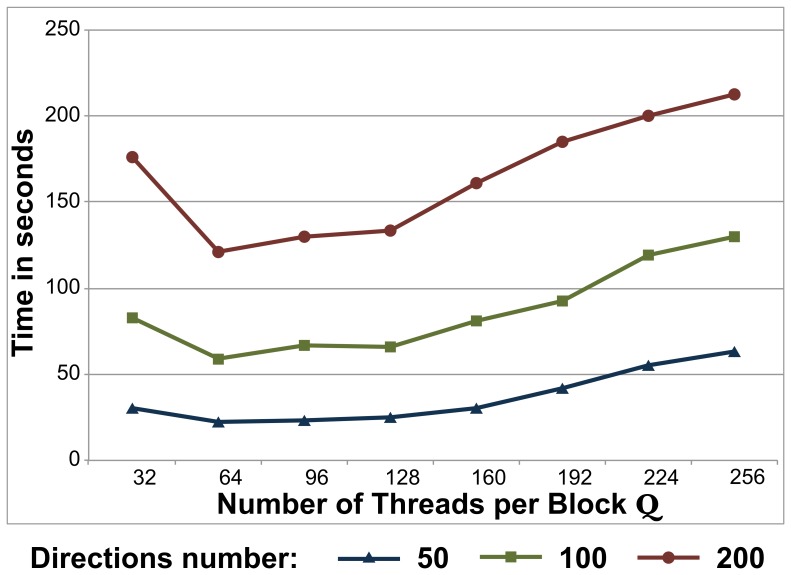
Execution times for the MCMC GPU kernel using different number of threads per block 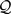. Results are shown for different number *K* of gradient directions (50, 100 and 200), for a slice of 4804 voxels (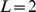 fibres, 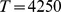 MCMC iterations (3000 burn-in)).

As in the case of the Levenberg-Marquardt kernel, when we choose the number of threads per block in the MCMC kernel, our target is to have simultaneously as many warps per SM as possible (to “hide” latencies from Global memory accesses). In the MCMC kernel, there are additional accesses, as the random numbers used during the MCMC are pre-generated and stored in the Global memory. If we use 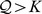 there will be idle threads inside the block and we will have unused resources (SPs and shared memory). If 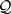 is not a multiple of *K* then the gradient directions will be distributed unevenly across threads. In addition, the limitations discussed in previous sections also exist, making the choice of the optimal 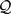 less straightforward. We therefore evaluated performance for different numbers of 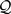 and different numbers of *K*. Figure 6 shows some illustrative examples. As it can be observed, a 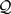 of 64 gave on average the smallest execution times. We therefore chose 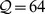 for this kernel.

### Multi-GPU Design

Up to now, we have exploited the hardware resources of a single GPU to speed-up bedpostX by using a granularity defined by the number of voxels and of diffusion-sensitising gradients. However, the intrinsic, higher-level parallelism using groups of voxels may be used to enhance even more the performance of the application in a multi-GPU environment. Therefore, we divide the voxels into a set of slices and assign each slice to one of the GPUs. A GPU runs both kernels for all voxels of the assigned slice. Such a design, suitable for running on a GPU cluster, is compared in the following sections to the CPU cluster design of FSL.

We should point out that, in general, cluster-based designs may require extra time to achieve communication across different nodes (GPUs or CPU cores). In our case, the different nodes don’t need to communicate because the slices are totally independent. Therefore, execution times reported for both multi-GPU and multi-CPU versions do not include any overheads of this type.

## Experiments

### Hardware Features

We have used two different GPU-based systems for testing our design. Both systems are Intel-based hosts that provide service to GPU devices. The first system had a Nvidia Tesla C2050 GPU [40] and a main processor Intel Xeon E5620 2.40 GHz with 16 GB of main memory. The second system was a GPU cluster with 372 Nvidia Tesla M2090 GPUs [41], based on the Fermi architecture [35]. It comprised of 84 processors Intel Xeon X5650 2.66 GHz, with 48 GB-96 GB of main memory each. The cluster used the platform LSF 8 [42] to handle jobs. Major features for the GPU devices are summarized in Table 1. Table 2 shows the theoretical peak performance in FLOPS (FLoating-point Operations Per Second) for each used device.

**Table 1:** Major Hardware features for Tesla C2050 and M2090 GPUs.

GPU element	Feature	Tesla C2050	Tesla M2090
SPs(GPU cores)	SPs per SM	32	32
	SMs	14	16
	Total num. cores	448	512
	Clock frequency	1.15 GHz	1.3 GHz
Maximumnumber ofthreads	Per SM	1536	1536
	Per block	1024	1024
	Per warp	32	32
SRAMmemoryavailableper SM	32-bit registers	32 K	32 K
	Shared memory	16/48 KB	16/48 KB
	L1 cache	48/16 KB	48/16 KB
	Total SRAM		
	(shared+L1)	64 KB	64 KB
	Size	3 GB	6 GB
Global(video)memory	Speed	2×1.546 GHz	2×1.85 GHz
	Width	384 bits	384 bits
	Bandwidth	148 GB/sec	177 GB/sec
	Technology	GDDR5	GDDR5
		DRAM	DRAM

**Table 2:** Theoretical Peak Performance of the GPUs devices and CPU cores used.

Device	Cores	ClockFrequency	SinglePrecision	DoublePrecision
NVIDIA Tesla C2050	448	1.15 GHz	1030.4 GFLOPS	515.2 GFLOPS
NVIDIA Tesla M2090	512	1.3 GHz	1331.2 GFLOPS	665.6 GFLOPS
1 core of Intel Xeon E5620	1	2.40 GHz	19.2 GFLOPS	9.6 GFLOPS
1 core of Intel Xeon X5650	1	2.66 GHz	21.28 GFLOPS	10.64 GFLOPS

Comparisons were performed using both GPU systems and their respective CPUs. Single-core experiments were performed on the first system, with the sequential version of the algorithm running on a single-core CPU and the CUDA design on the GPU. Multi-GPU and multi-CPU experiments were performed on the cluster, using respectively as many GPUs and CPU cores as the number of slices in the dataset.

Finally, we used for our tests the 4.2 CUDA compiler version (also 4.2 Driver Version and 4.2 Runtime Version) and the gcc 4.4.3 compiler version.

### Diffusion-weighted MRI Data

A healthy male subject was scanned in a 3 T Siemens Trio clinical imaging system, after giving informed consent. A diffusion-weighted acquisition (single-shot EPI) was performed. The acquisition matrix was 96×96 with in-plane resolution 2×2 mm^2^ and 2 mm slice thickness (TR = 4.9 s, TE = 111 ms, 32-channel coil, 6/8 partial Fourier). Thirty slices were acquired in total and diffusion weighting was applied in 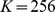 evenly spaced directions with b = 2500 s/mm^2^.

Another high-resolution dataset was acquired using a 3 T Connectom Skyra system. A diffusion-weighted acquisition (single-shot EPI) was performed, with an acquisition matrix of 140×168, in-plane resolution 1.25×1.25 mm^2^ and 1.25 mm slice thickness (TR = 4.8 s, TE = 85 ms, 32-channel coil, 6/8 partial Fourier). A hundred and two slices were acquired in total and diffusion weighting was applied in 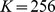 evenly spaced directions with b = 1500 s/mm^2^. A multiband factor of 3 was employed [25], [43].

We used the above datasets to compare the computational performance of the two designs, sequential and CUDA. Tests were performed for different number *R* of model parameters, which increase with *L*, the number of fibres in the ball & stick model. Also, for different number of diffusion-sensitising gradients *K*, by taking subsets from the original dataset. The first dataset was used for all the comparisons between a single-core CPU and a GPU. The high resolution dataset was used to compare the multi-GPU and multi-CPU versions.

## Results

Before comparing the different designs in terms of their execution time, we first evaluated the similarity of their model estimates. Due to the random aspects of the MCMC algorithm, each execution of the algorithm returns slightly different model estimates; even if the same design is ran on the same data. However, model estimates for a given design are expected to be the same *on average*. For that reason, we chose some representative voxels in the brain, from regions with different anatomical features, and we examined whether the different designs return on average the same results. For every voxel, both CPU and GPU designs were executed 1000 times each. For each execution, the mean of the respective posterior distribution for every model parameter was recorded. The histograms of these mean values across the 1000 repeats were then compared for the CPU and GPU design. Three examples are shown in Figure 6 that also show the histograms of three different model parameters. It is clear that both designs return almost identical estimates.

A second way of validating the CUDA design against the sequential one is to store and keep all random numbers exactly the same for both designs and compare the MCMC estimates. We performed this second test (results not shown) and we found differences only due to precision caused by the different rounding modes between CPU and GPU [44].

We further performed performance comparisons and assessed speed-ups achieved by the CUDA design, under different aspects:

- Comparison of each CUDA kernel versus its CPU counterpart.
- Scalability of performance when varying the number of iterations for each kernel.
- Scalability when increasing the number *R* of model parameters, the dataset size (number of voxels) or the number *K* of diffusion-sensitising gradient directions.
- Overall comparison of single GPU versus the single-core CPU counterpart version included in FSL.
- Comparison of our multi-GPU version versus a multi-CPU version using a cluster.

### Levenberg-Marquardt kernel evaluation

**Figure 7:**
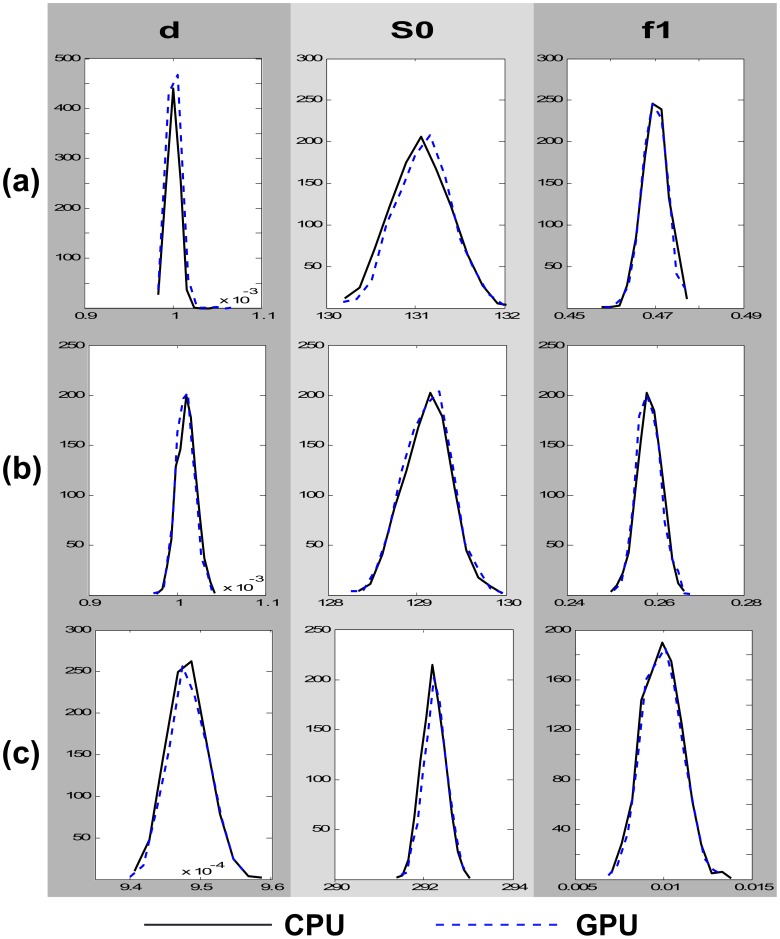
Comparison between CPU and GPU model estimates for the diffusivity *d*, the baseline signal 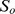 and the volume fraction of the first fibre 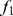, in different brain areas. (a) A corpus callosum voxel, (b) a centrum semiovale voxel and (c) a grey matter voxel. Each design was ran 1000 times on the same data and for each repeat the mean of the posterior distribution of the respective parameter was recorded. The histograms show the distributions of these means across all 1000 repeats. For each repeat, a burn-in period of 3000 iterations and a thinning period of 25 samples was used for the MCMC.

Figure 7 shows a performance comparison between a single-core CPU and a GPU when executing Levenberg-Marquardt for three data sets with different number *K* of gradient directions (64, 128 and 256) and when estimating 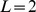 fibres. In Figure 7a execution times for a slice are shown as the number of iterations is increased (i.e. the convergence criterion for the optimisation is reduced). A good scalability of the GPU version is shown compared to the CPU version. In Figure 7b, execution times are shown for different slice sizes (i.e. different number of voxels to be processed). A linear speedup is obtained whenever the number of voxels increases. The maximum speed-up for 128 gradient directions was 120x.

### MCMC kernel evaluation

**Figure 8:**
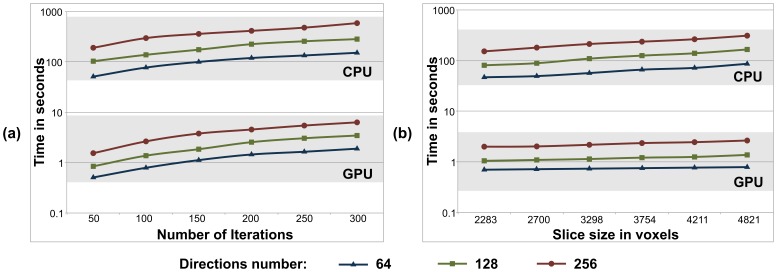
Comparison of single-core CPU and GPU execution times (in log scale) running the Levenberg-Marquardt algorithm with speed gains over two orders of magnitude: (a) As the number of Levenberg-Marquardt iterations are increased, and (b) as the number of voxels per slice are increased. The execution times for (a) are for a slice of 4804 voxels, with the convergence criterion of the algorithm decreased to allow more iterations. For each case, results are shown for different number *K* of gradient directions (64, 128 and 256) and for estimating 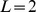 fibres.

Figure 8 shows the performance evaluation of the MCMC kernel on a single GPU compared to the sequential counterpart version in a single-core CPU for three data sets with different number *K* of gradient directions (64, 128 and 256) and for estimating 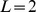 fibres. Similar as before, Figure 8a presents execution times as the number of MCMC iterations increase and Figure 8b as the number of voxels to be processed increase. In both cases, we can see the scalability of the GPU version versus the CPU version. The maximum speed-up for 128 gradient directions was 135x.

### Overall performance in a single GPU

**Figure 9:**
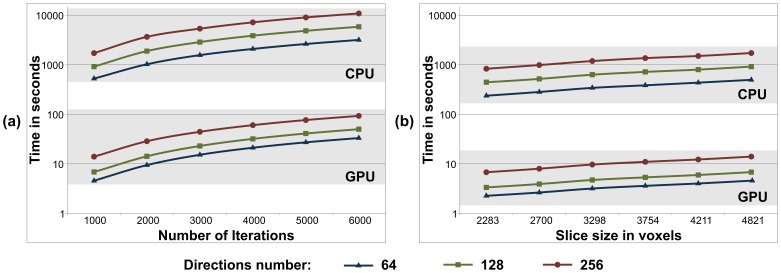
Comparison of single-core CPU and GPU execution times (in log scale) running the MCMC algorithm with speed gains over two orders of magnitude: (a) As the number of MCMC iterations are increased, and (b) as the number of voxels per slice are increased. The execution times for (a) are for a slice of 4804 voxels and for (b) for 1000 MCMC iterations. For each case, results are shown for different number *K* of gradient directions (64, 128 and 256) and for estimating 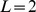 fibres.

The total execution times of the bedpostX application on a single-core CPU and a GPU are shown in Figure 9 for processing the whole dataset (30 slices). Execution times are plotted against the number of fibres *L* to be estimated and for different number *K* of gradient directions. Table 3 summarizes the speed-up factors obtained for bedpostX.

**Table 3:** Speed-ups for running bedpostX in a GPU over a single-core CPU.

*K* GRADIENT DIRECTIONS	*L* FIBRES	SPEEDUP
	1	68x
64	2	77x
	3	79x
	1	95x
128	2	95x
	3	96x
	1	112x
256	2	99x
	3	97x

### Overall Performance in a Multi-GPU System

**Figure 10:**
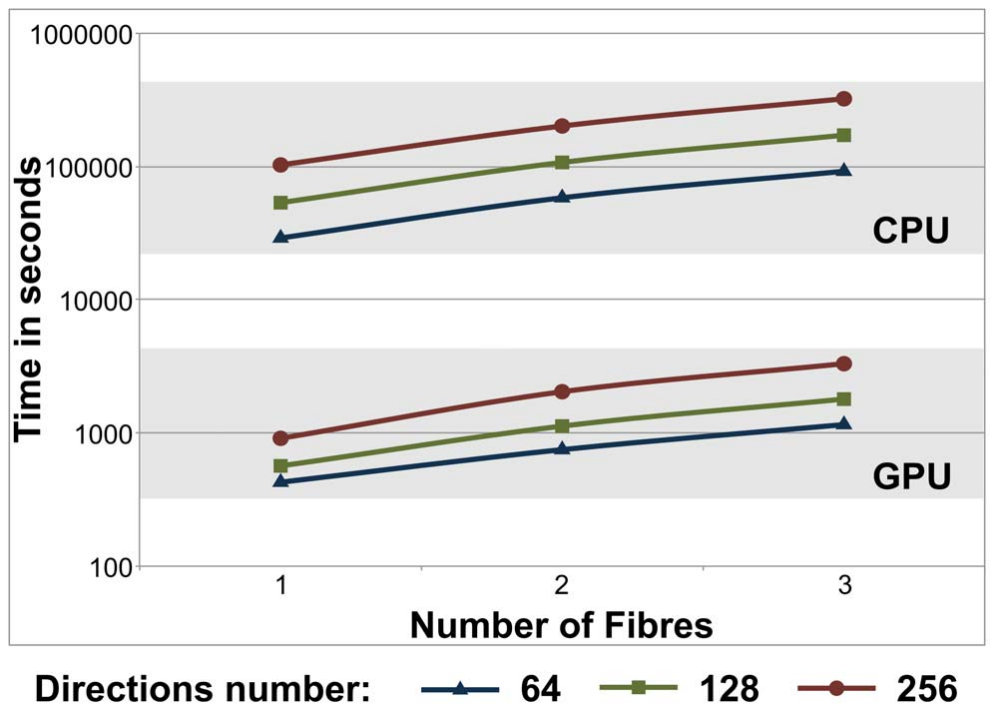
Total execution times (in log scale) of the bedpostX application in a single-core CPU and a Tesla C2050 GPU for the whole dataset (30 slices), as the number of fibres *L* is increased. Results are shown for different number *K* of gradient directions (64, 128 and 256) and when 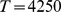 MCMC iterations were utilised (3000 burn-in iterations).

Figure 10 shows the execution times of bedpostx in a cluster of either CPUs or GPUs. The reported times are for the high-resolution dataset of 102 slices as the number of fibres *L* is increased. The experiments were performed in the supercomputer previously described in Section. Concretely, we used 102 CPU cores and 102 GPUs. Table 4 summarizes the speed-up factors obtained for bedpostX.

**Table 4:** Speed-ups for running bedpostX in a cluster of GPUs over a cluster of CPUs.

*K* GRADIENT DIRECTIONS	*L* FIBRES	SPEEDUP
	1	71x
64	2	85x
	3	86x
	1	88x
128	2	108x
	3	109x
	1	104x
256	2	116x
	3	120x

## Discussion

We have designed and implemented a GPU-based parallelisation of a popular toolbox that is used for brain anatomical studies. The bedpostX tool within the FSL software is commonly used to estimate fibre orientations from diffusion-weighted magnetic resonance images. Our design included the implementation of two main CUDA kernels, which we validated and compared against single-core CPU counterparts. We have achieved speed-up factors of up to 112x in a single GPU compared to its sequential single-core CPU counterpart version. We also tested the comparison of our design in a multi-GPU system and achieved a 120x speed-up to a multi-CPU version. These improvements mean, in practical terms, that our approach reduces the processing time of bedpostX to a few minutes per subject using a single GPU on commonly acquired diffusion MRI data (2 mm isotropic, whole brain coverage, 60 diffusion-sensitising directions).

**Figure 11:**
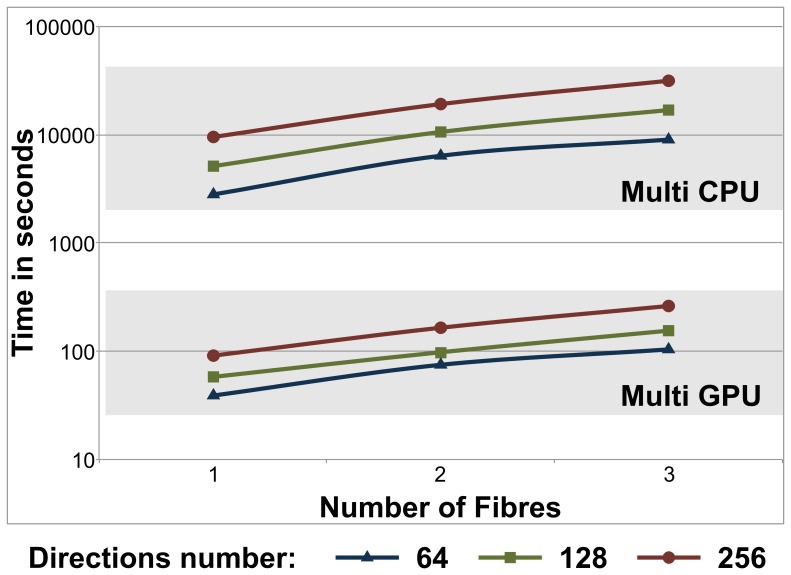
Total execution times (in log scale) of the bedpostX application in a CPU cluster and 372 GPUs Tesla M2090 processing 102 slices, as the number of fibres *L* is increased. Results are shown for different number *K* of gradient directions (64, 128 and 256) and when 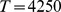 MCMC iterations were utilised (3000 burn-in iterations).

The scalability in both the single and multi-GPU/CPU architectures is roughly steady, in number of GPUs/CPU cores (Figures 10, 11). We can therefore extrapolate execution times and compare performances of configurations that were not explicitly tested here. For instance, if we would like to compare the single-GPU version with a CPU cluster, we would expect that we need more than 100 cores on the cluster to achieve a similar performance as a single GPU, but of course at a multiple cost.

Although we have obtained a notable reduction in execution time, the CPU is only monitoring the execution of the GPU in the current implementations. We can also use the CPU to compute part of the workload, taking advantage of the heterogeneous nature of the modern computers and this will be part of a future study.

The CUDA programming model is a proprietary closed platform. OpenCL (Open Computing Language) [45] is an open standard and an alternative for these implementations. At this stage, we opted for CUDA, as it is currently more mature and achieves better performance [46]. However, our design is not particularly tied to CUDA and its main components could be implemented using a different platform.

The computational performance reported in this study cannot be underestimated. Efforts to map the structural connections in the human brain, such as the Human Connectome Project [25], will provide massive and high-resolution datasets, whose processing is very computationally demanding. Furthermore, advances in MRI protocol acceleration, such as multiband acquisition [43], will allow the collection of much more data at a given amount of time. Input datasets from future acquisitions will be much larger in size and much more demanding for their processing. Therefore, speed-ups of more than two orders of magnitude in data processing, as the ones reported here, are heavily beneficial.

Other studies have recently proposed the utilisation of GPUs in the context of brain connectivity analysis and tractography. However, in [47], [48] GPUs were used only for visualising tractography results. The approaches proposed in [49]–[51] perform deterministic tractography rather than dealing with a probabilistic Bayesian inference framework, as in our study. Finally, Xu et al have very recently presented an implementation with a similar aim to ours [52]. However, their MCMC GPU-based design achieves a maximum speed-up of 34x compared to the single-core CPU version, significantly smaller than the 112x speed-up that our design achieved. Even if it is difficult to identify, we believe that this difference is mostly due to our MCMC kernel design (Figure 5) and the second level of parallelisation achieved.
