# Correction: Accelerating Fibre Orientation Estimation from Diffusion Weighted Magnetic Resonance Imaging Using GPUs

**DOI:** 10.1371/journal.pone.0130915

**Published:** 2015-06-12

**Authors:** Moisés Hernández, Ginés D. Guerrero, José M. Cecilia, José M. García, Alberto Inuggi, Saad Jbabdi, Timothy E. J. Behrens, Stamatios N. Sotiropoulos

The in-text citations for Figures 6–10 are incorrect. The in-text citations to Figure 6 should be to Figure 7. The in-text citations to Figure 7 should be to Figure 8. The in-text citations to Figure 8 should be to Figure 9. The in-text citations to Figure 9 should be to Figure 10. The in-text citations to Figure 10 should be to Figure 11.
